# Extraction of Lipophilic Antioxidants from Native Tomato Using Green Technologies

**DOI:** 10.17113/ftb.60.01.22.7366

**Published:** 2022-03

**Authors:** Darío R. Gómez-Linton, Arturo Navarro-Ocaña, Silvestre Alavez, Ricardo Lobato-Ortiz, Angélica Román-Guerrero, José Alberto Mendoza-Espinoza, Juan Manuel Villa-Hernández, Laura J. Pérez-Flores

**Affiliations:** 1 Biotechnology Ph.D. Program, Metropolitan Autonomous University, Iztapalapa Campus, F.F. C.C. St. Rafael Atlixco Ave. 186, 09310, Mexico City, Mexico; 2 National Autonomous University of Mexico, Outer Circuit, Coyoacan, 04510, Mexico City, Mexico; 3 Metropolitan Autonomous University, Lerma Campus, Herons Ave. 10, 52005, Lerma de Villeda, Mexico State, Mexico; 4 Postgraduate College, Montecillo Campus, Mexico-Texcoco Street km 36.5, Texcoco, 56230, Mexico State, Mexico; 5 Metropolitan Autonomous University, Iztapalapa Campus, F.F. C.C. St. Rafael Atlixco Ave. 186, 09310, Iztapalapa, Mexico City, Mexico; 6 Autonomous University of Mexico City, Liberty House Campus, Ermita Iztapalapa Road 4163, 09620, Iztapalapa, Mexico City, Mexico.; § Current affiliation: University of the Sea, Km 1.5 road to Sola de Vega, 71980, Puerto Escondido, Oaxaca, Mexico

**Keywords:** lipophilic antioxidants, native tomato genotype, enzyme-assisted extraction, sonication, green solvents

## Abstract

**Research background:**

Tomato (*Solanum lycopersicum* L.) fruit is highly consumed worldwide and contains high amounts of carotenoids and tocopherols, two powerful antioxidants. Native tomato genotypes are rarely used in large-scale market but serve as a reservoir to diversify the species gene pool and can be employed to obtain functional compounds. Extraction methods are currently changing towards cleaner procedures that are more efficient and environmentally friendly, including avoiding toxic or polluting solvents.

**Experimental approach:**

In this study, factorial and fractional factorial designs were used to evaluate the efficiency of digestive enzymes, sonication and green solvents to obtain lipophilic antioxidant extracts from native tomato. To monitor the efficiency of the extraction process, spectrophotometric quantification of total carotenoids and antioxidant activity was carried out, and then individual quantification of carotenoids and tocopherols in the extracts was done by HPLC.

**Results and conclusions:**

Digestive enzymes and sonication increased the carotenoid content and the antioxidant activity of the obtained extracts when applied individually. However, when these treatments were applied together and in combination with isopropyl acetate, a green solvent, the obtained extracts had the highest carotenoid and tocopherol contents as well as the maximal antioxidant activity. A correlation analysis suggested that antioxidant activity resulted from synergistic effects rather than individual compounds. Tomato extracts were obtained through a rapid and environmentally friendly extraction method and their antioxidant activity was enhanced.

**Novelty and scientific contribution:**

Tomato fruits have been the subject of numerous studies; however, functional compound extraction through environmentally friendly methods remains an attractive use of native tomato fruit, enhancing its limited production and harnessing a large amount of tomato product industry. There are few reports where environmentally friendly extraction methods are combined; even rarer are those where green solvents are also used. In this work, the combination of different environmentally friendly extraction methods improved the extraction of carotenoids and tocopherols and allowed to establish a more efficient process. These results could stimulate the use of clean technologies and make the native tomato more attractive for industrial or compound extraction processes.

## INTRODUCTION

Tomato (*Solanum lycopersicum* L.) is a fruit native to South America (*1*). It is the second most important crop in the world (*2*) and a significant source of antioxidants in the human diet due to its high consumption (*3*, *4*). The main hydrophobic antioxidants in tomatoes are lycopene, β-carotene and α-tocopherol, while vitamin C and polyphenolic compounds (such as quercetin, kaempferol, naringenin and rutin) are the main hydrophilic functional compounds (*5*, *6*). It has been reported that native tomato genotypes (tomato landraces) have higher contents of functional compounds such as lycopene or polyphenols, as well as greater *in vitro* antioxidant capacity than commercial varieties (*7*, *8*). Hence, native tomatoes have the potential to be incorporated into genetic improvement programmes or to be used at the commercial and industrial levels.

Reactive oxygen species play a role in the development of several degenerative diseases, including cancer, diabetes, cardiovascular and inflammatory diseases, neuronal degeneration and ageing (*9*, *10*). Some studies have linked tomato consumption to a lower incidence of these diseases (*11*-*13*), this association may be mediated by antioxidants (*14*, *15*).

Developing adequate and sustainable methods for extracting functional compounds from plant matrices is an area of great potential; ideally, these methods should be environmentally friendly and safe (*16*). The use of these compounds is of interest to industries such as food, cosmetics and pharmaceuticals, among others. Because many functional compounds are prone to degradation when isolated from their original sources, the extraction method should be selected or developed to reduce the possible stages of degradation (*17*). Some simple non-conventional methods like ultrasound and lytic enzymes enhance extraction efficiency compared to traditional ones (*18*, *19*). In addition, the use of green solvents, which are more friendly and safer than conventional solvents, is highly recommended (*20*).

Different experimental designs, applied to the extraction processes, provide a better understanding of the effect of parameters related to yield (time, temperature, solvent, *etc*.), making them powerful tools for researchers. In recent years, the use of incomplete designs such as a fractional factorial design has increased because they provide valuable information with a relatively low resource investment (*21*). This study aims to optimise the extraction of functional compounds from native tomato by using hydrolytic enzymes, sonication and green solvents to achieve a higher yield in an environmentally friendly manner.

## MATERIALS AND METHODS

### Biological samples

Saladette commercial tomatoes at consumption maturity were acquired from a local market in Iztapalapa, Mexico City, Mexico, and used to perform experiments 1 and 2. Samples for experiment 3 were two native tomato genotypes (cherry, code 209, ID LOR88, collected in Teotitlán de Flores Magón, Oaxaca, Mexico [18°07'57"N 97°04'20"W] and ojo de venado (deer’s eye), code 210, ID LOR118, collected in La Ceiba, Puebla, Mexico [20°23'N, 97°52'W]) (*22*) collected and donated by Ricardo Lobato PhD. For their study, the tomato plants were cultured at the Postgraduate College, Montecillo, Texcoco, Mexico (19°30'N, 98°53'W) in 2018. Both native genotypes used in this study are part of the Network of Tomato (Red de Tomate, Subcomité de Recursos Genéticos Agrícolas, Secretaría de Agricultura y Desarrollo Rural, Mexico City, Mexico).

### Obtaining lipophilic tomato extracts

Three experiments, denoted as experiments 1, 2 and 3 were carried out for obtaining the extracts. In experiment 1, the application of digestive enzymes was tested by using a factorial design with two factors, the enzymatic cocktail and the reaction medium. Subsequently, experiments 2 and 3 comprised fractional factorial designs (2^4-1^). In experiment 2, the reaction medium, reaction time, enzyme cocktail concentration and reaction temperature were studied. In experiment 3, the evaluated factors were the tomato genotype, sonication application, enzyme cocktail application and green solvent.

#### Experiment 1: enzyme cocktail and reaction medium tests

The tested enzyme cocktails were NS-22002/CNN02196 (CNN) with glucanase, xylanase, hemicellulase and cellulase activities; NS-50012/KTN02163 (KTN) with cellulase, glucanase, hemicellulase, pectinase and xylanase activities; and Viscozyme L (VIS) with hemicellulase, glucanase, cellulase, arabanase and xylanase activities. These enzymes were obtained from *Humicola insolens*, *Aspergillus aculeatus* and *Aspergillus* sp., respectively, and were acquired from Novozymes (Mexico City, Mexico). The enzymatic treatments were carried out in 0.2 M acetate buffers (pH=4, 5 or 6; sodium acetate-acetic acid, J.T. Baker, Xalostoc, Mexico) or distilled water to test the enzymatic reaction at unregulated pH. The added enzyme cocktail was 5 mL per 100 g fresh mass of tomato fruit.

Briefly, 1 g of commercial saladette tomato pulp was frozen and pulverized in a mortar with liquid N_2_. After that, it was mixed with 3 mL of one of the enzymatic reaction media. Subsequently, the enzyme cocktail was added and the mixture was kept under constant stirring (300 rpm) in an incubator (Incubator Shaker II, model 136400; Boekel Industries Inc., Feasterville-Trevose, PA, USA) for 3 h at 40 °C in the dark. Afterwards, the tomato pulp was separated from the reaction medium by filtration. The residue was washed with 10 mL distilled water and extracted with 10 mL dichloromethane (J.T. Baker) by vortexing (Genie II SI-0236, Scientific Industries, Bohemia, NY, USA) for 15 min at medium intensity. Then, 4 mL ethanol (J.T. Baker) and 6 mL distilled water were added and the mixture was centrifuged at 4500×*g* for 10 min (Avanti J-30I; Beckman Coulter Inc., Brea, CA, USA). The aqueous and organic phases were collected separately; the organic phase was filtered through 0.45 µm nylon membranes (Merck Millipore Corporation, Burlington, MA, USA) and adjusted to 10 mL with dichloromethane. The product was stored at -70 °C (ultra-low temperature freezer Forma 88400V; Thermo Fisher Scientific, Waltham, MA, USA) until analysis.

#### Experiment 2: enzymatic reaction conditions

Four factors were studied to evaluate the enzymatic reaction conditions, including reaction medium (-1: pH=5, 1: distilled water), reaction time (-1: 1 h, 1: 5 h), enzyme cocktail amount (KTN, -1: 1 mL/100 g, 1: 5 mL/100 g) and temperature (-1: 50 °C, 1: 40 °C) (Table 1). The studied factor levels were selected by preliminary assays or following recommendations in the literature (*18*, *19*, *23*-*25*). The treatment arrangement was generated by the Statgraphics Centurion XVI software, v. 16.2.04 (*26*). Briefly, 1 g commercial saladette tomato pulp was frozen with liquid N_2_ and pulverised in a mortar. The powder was mixed with 3 mL of distilled water or 0.2 M acetate buffer (pH=5). The tested amount of enzyme cocktail (10 or 50 µL) was added and incubated with constant stirring for 1 or 5 h at 40 or 50 °C, depending on the experimental design. Once the incubation was over, the extraction was carried out following the method applied in experiment 1.

**Table 1:** Experimental design for experiment 2: enzymatic reaction conditions

Treatment	Reaction medium (-1=pH, 1=DW)	Time/h (-1=1, 1=5)	(*V*(enzyme)/*m*(fruit))/(mL per 100 g) (-1=1, 1=5)	*Temperature/°C (-1=50, 1=40)
1	-1	-1	-1	1
2	1	1	1	-1
3	-1	1	-1	-1
4	1	-1	1	1
5	-1	-1	1	-1
6	1	1	-1	1
7	-1	1	1	1
8	1	-1	-1	-1

*Complement=-(A·B·C). pH=5, DW=distilled water

#### Experiment 3: non-conventional extraction methods

For the combination of non-conventional methods, two genotypes of native tomatoes (209 and 210) were studied. The other three factors studied were sonication (-1: no, 1: yes), KTN cocktail (-1: no, 1: yes) and green solvent (-1: isopropyl acetate, 1: ethyl lactate); the treatment arrangement was generated by the Statgraphics Centurion XVI software, v. 16.2.04 (*26*) (Table 2).

**Table 2:** Experimental design for experiment 3: combination of environmentally friendly extraction methods

Treatment	Genotype -1=209, 1=210	Sonication -1=no, 1=yes	Enzyme -1=no, 1=yes	*Solvent -1=IA, 1=EL
1	1	1	1	1
2	1	-1	-1	1
3	-1	1	1	-1
4	-1	-1	-1	-1
5	1	1	-1	-1
6	1	-1	1	-1
7	-1	1	-1	1
8	-1	-1	1	1

*Complement=(A·B·C). IA=isopropyl acetate, EL=ethyl lactate

The procedure was as follows: 0.5 g tomato was frozen and pulverised in a mortar with liquid N_2_. Subsequently, the tomato was mixed with 5 mL of *φ*(ethanol)=20%, vortexed for 5 min and centrifuged (Avanti J-30I; Beckman Coulter Inc.) at 4500×*g* for 10 min. The supernatant was separated and 2 mL of 0.2 M acetate buffer (pH=5) were added to the plant residue. According to the experimental design, the enzyme (5 mL/100 g) was added and incubated for 1 h at 50 °C with stirring (300 rpm). Subsequently, the samples were centrifuged at 4500×*g* and the plant material was separated by filtration, recovered and washed with 5 mL distilled water. In cases where sonication was applied, the pellet was mixed with 5 mL of the solvent (isopropyl acetate or ethyl lactate; Sigma-Aldrich, Merck, St Louis, MO, USA) and sonicated for 10 min with an ultrasonic probe (Vibra-Cell™, 130 W, 20 kHz; Sonics and Materials Inc., Newtown, CT, USA) in an ice bath. It was subsequently centrifuged at 4500×*g* for 10 min and the organic phase was collected for analysis. In the treatments where sonication was not applied, after enzymatic treatment and subsequent recovery of the plant material, 5 mL solvent were added to the tomato, vortexed for 10 min and centrifuged at 4500×*g*. The organic phase was collected and stored at -70 °C until analysis.

### Determination of antioxidant activity

The method reported by Re *et al.* (*27*) was followed. A solution of 2,2′-azino-bis(3-ethylbenzothiazoline-6-sulfonic acid) diammonium salt (ABTS) radical (Sigma-Aldrich, Merck) was prepared with 16.5 mg potassium persulfate (J.T. Baker) and 96.2 mg ABTS in 100 mL of distilled water. An aliquot from this solution was taken and diluted in *φ*(ethanol)=96% until it reached an absorbance of 0.7 at 734 nm. A volume of 100 μL of the sample was mixed with 1000 µL of ABTS solution, incubated in the dark for 10 min, and the absorbance was read using a spectrophotometer (DU650 spectrophotometer; Beckman Coulter Inc.) at 734 nm. Quantification was performed using a Trolox standard (Sigma-Aldrich, Merck) curve and results are expressed in µmol Trolox equivalents (TE) per gram of fresh mass (fm).

### Determination of total carotenoid content by spectrophotometry

The total carotenoid content in tomato extracts was measured by spectrophotometric readings at an adequate dilution to obtain an absorbance between 0.25 and 0.85. Dilutions were made with dichloromethane, which was used as the blank. The residue of other solvents did not affect the scan between 350 and 750 nm. The measurement was carried out at 482 nm using a molar absorption coefficient *ε*=248.0 L/(g·cm), which is reported for lycopene in dichloromethane (*28*). The results are expressed as µmol lycopene equivalents per g fm.

### Determination of the mass fraction of individual carotenoids by high-performance liquid chromatography

The technique reported by Fraser *et al.* (*29*) was followed with some modifications. The samples were injected into an 1260 Infinity II high-performance liquid chromatograph (HPLC; Agilent Technologies, Santa Clara, CA, USA) with a quaternary pump and autosampler. A Waters Xterra MS C18 column (5 μm, 4.6 mm×250 mm; Waters Corporation, Milford, MA, USA) at 25 °C was used with acetonitrile/methanol/dichloromethane (43:43:14 *V*/*V*/*V*; J.T. Baker) as the mobile phase in an isocratic run. The flow was 1 mL/min and the detector (diode-array detection) was fixed at 455 nm. Quantification was performed by using standard curves of lycopene, β-carotene and lutein (Sigma-Aldrich, Merck). The results are expressed in µg carotenoid per g fm.

### Determination of the mass fraction of individual tocopherols by high-performance liquid chromatography

The tocopherol content of the extracts was quantified according to the method of Mène-Saffrané *et al.* (*30*) with the following modifications. An HPLC system (Shimadzu Prominence 20; Shimadzu Corporation, Kyoto, Japan) equipped with a Shimadzu RF-20 fluorescence detector and a LiChrospher 100 Diol column (4.6 mm×250 mm, 5 μm; Merck Millipore Corporation) was used. The mobile phase was hexane/methyl *tert*-butyl ether, 90:10 (J.T. Baker) in an isocratic run at 0.8 mL/min flow rate. The wavelengths used were 296 nm for excitation and 340 nm for emission. For the identification and quantification, standard curves of α-, β-, γ- and δ-tocopherol (Sigma-Aldrich, Merck) were used. The results are expressed in µg tocopherol per g fm.

### Statistical analysis

**Fig. S1:**
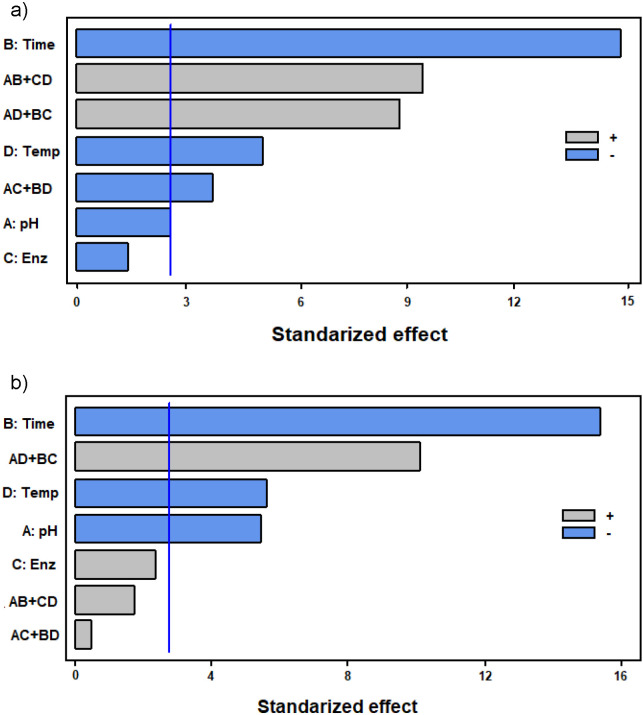
Pareto diagrams of: a) total carotenoid content, and b) antioxidant activity analyses corresponding to experiment 2. A=pH, B=time, C=enzyme *V* (mL enzyme per 100 g), D=temperature. The bars that exceed the horizontal blue line correspond to the factors that are significant. The symbols + | - indicate which factor level gives the best result, -1 or 1 (relevant only to significant individual factors). Terms such as AB+CD indicate an interaction between factors that the method is not able to discern, which limits the significance of these terms

**Fig. S2:**
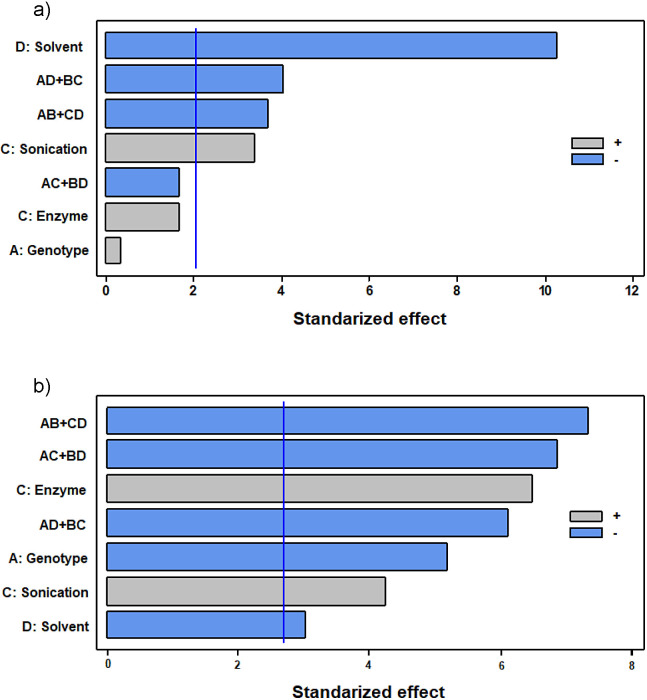
Pareto diagrams of: a) total carotenoid content, and b) antioxidant activity analyses corresponding to experiment 3. A=genotype, B=sonication, C=KTN enzyme cocktail, D=solvent. The bars that exceed the horizontal blue line correspond to the factors that are significant. The symbols + | - indicate which factor level gives the best result, -1 or 1 (relevant only to significant individual factors). Terms such as AB+CD indicate interaction between factors that the method is not able to discern, which limits the significance of these terms

The experimental designs and the data analysis were performed using the Design of Experiment (creation of new design and analyse design, respectively) tool of Statgraphics Centurion XVI, v. 16.2.04 software (*26*). For fractional factorial designs (2^4-1^), the software sets the level values (negative or positive) for the first three factors (A, B and C) and the fourth (complement) is set by the analyser as the positive or negative product of the previous three ((A·B·C) or -(A·B·C)). The results were analysed by analysis of variance (ANOVA) followed by Tukey’s test. Three replicates were made for each experiment. Significance was fixed at α=0.05. Pareto diagrams (Fig. S1 and Fig. S2) were also obtained with the analysis design tool.

## RESULTS AND DISCUSSION

### Effect of enzyme cocktails and reaction medium on extraction efficiency

Enzymatic treatment of different vegetal materials has been reported to be an effective way to improve extraction efficiency of various compounds of interest (*23*, *31*). We first evaluated whether the enzymatic treatment had significant effects on carotenoid content and antioxidant activity of the tomato extracts compared to a control sample without enzymatic treatment. At the same time, we determined which enzyme cocktail and reaction medium had the greatest benefits because enzymes are susceptible to modifications in their structure, activity or affinity to their substrate depending on the environment in which they are immersed (*16*, *32*).

Although the reported lytic activity of the different cocktails is similar, the specific composition of each one as well as the source of the obtained enzymes are different, so particularities such as optimal conditions for their activity and affinity with the substrates affected their performance in the tests. For this experiment, the reaction time and temperature were fixed at 3 h and 40 °C, respectively.

**Fig. 1:**
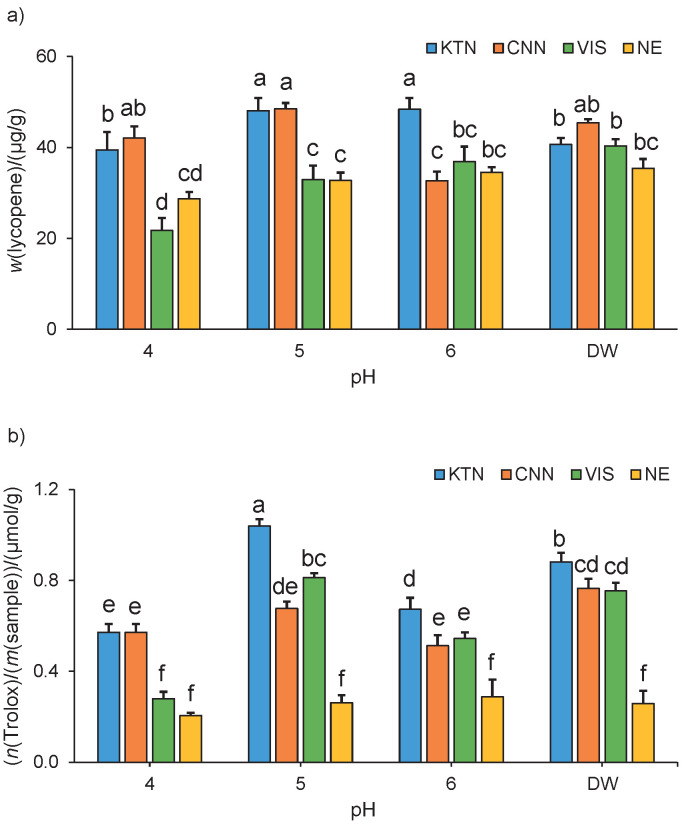
The average values (mean value±S.D.; *N*=3) of: a) carotenoid mass fractions, and b) antioxidant activities in commercial tomato extracts using different enzyme cocktails in different reaction media. Different letters indicate significant difference according to the Tukey’s test (α=0.05). DW=distilled water, KTN=enzyme cocktail with cellulase, glucanase, hemicellulase, pectinase and xylanase, CNN=enzyme cocktail with glucanase, xylanase, hemicellulase and cellulose, VIS=enzyme cocktail with hemicellulase, glucanase, cellulose, arabanase and xylanase, NE=no enzyme

Fig. 1 shows the average values of carotenoid mass fraction and antioxidant activity in the extracts obtained with the enzymatic treatments in the different reaction media tested. Both factors, reaction medium and enzyme cocktail, as well as their interaction significantly influenced (p<0.001) the carotenoid mass fraction (expressed as lycopene) and the antioxidant activity of the extracts. The KTN cocktail exhibits high performance in terms of carotenoid extraction as well as the maximum value of the antioxidant activity, with a significant difference compared to the control sample without enzyme. Concerning pH, when the reactions were carried out at pH=5, the average values of carotenoid mass fraction and antioxidant activity were the highest when using the KTN cocktail, a finding that is consistent with the previous reports for this cocktail (*24*). Recently, Ladole *et al.* (*19*) reported that pH=5 is optimal for tomato lycopene extraction using a pectinase-cellulase enzyme mixture. Interestingly, among the enzyme cocktails tested in this work, the KTN cocktail is the only one with pectinases (*24*, *33*, *34*), which might account for the obtained results. Enzymes can increase the yield of carotenoids in tomato extracts because these pigments are accumulated in specialised intracellular organelles and are not readily reached by solvents (*19*). In earlier reports, better yields of tomato carotenoids were reported by using pectinases (alone or in combination with cellulase) for enzyme-assisted lycopene extraction from tomato fruit and waste than by enzymatic treatments without pectinase (*25*, *31*).

Surprisingly, the treatment with the KTN cocktail using distilled water as the reaction medium had the second-highest values of carotenoid content and antioxidant activity. This result is interesting because it suggests that the external regulation of the pH of the enzymatic reaction might not be mandatory to obtain good results, perhaps due to the composition of tomato extracts. This would be an advantage for economical and simplicity reasons. Both factors, enzyme and reaction medium, as well as their interaction were significant. It was, therefore, necessary to study them in a more detailed experiment. Based on these results, the KTN enzyme cocktail and two media, the acetate buffer (pH=5) and distilled water were selected for use in experiment 2.

### Optimisation of the enzymatic reaction conditions

**Fig. 2:**
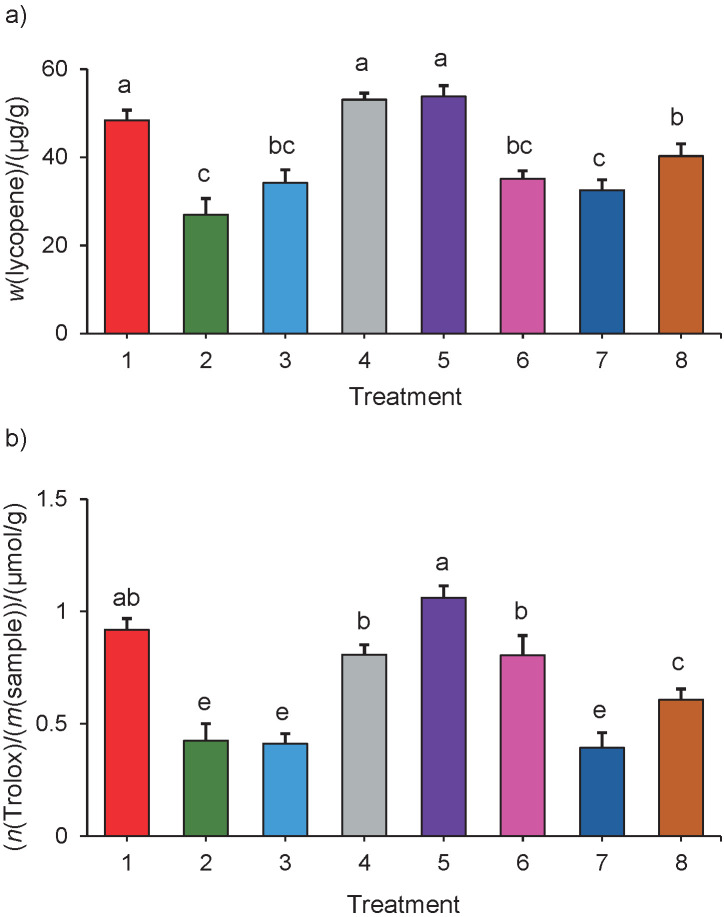
The average values of: a) carotenoid mass fractions, and b) antioxidant activities of extracts obtained using the KTN enzyme cocktail (with cellulase, glucanase, hemicellulase, pectinase and xylanase activities) under different enzymatic reaction conditions (1: pH=5, 1 h, 1 mL per 100 g, 40 °C; 2: distilled water, 5 h, 5 mL per 100 g, 50 °C; 3: pH=5, 5 h, 1 mL per 100 g, 50 °C; 4: distilled water, 1 h, 5 mL per 100 g, 40 °C; 5: pH=5, 1 h, 5 mL per 100 g, 50 °C; 6: distilled water, 5 h, 1 mL per 100 g, 40 °C; 7: pH=5, 5 h, 5 mL per 100 g, 40 °C; 8: distilled water, 1 h, 1 mL per 100 g, 50 °C.). Different letters indicate significant difference according to the Tukey’s test (α=0.05)

Fractional factorial experiments have the advantage of allowing the incorporation of qualitative factors (*35*); the reaction medium corresponds to a qualitative factor, while enzyme amount, time of enzymatic reaction and temperature of reaction are quantitative factors. In addition to the pH of the medium, which was partially tested in the initial factorial experiment, the temperature, enzyme amount and reaction time are factors that can affect the effectiveness of the enzyme activity (*23*, *32*). The results of experiment 2 are given in Fig. 2.

Extracts obtained by treatments 1 (pH=5, time=1 h, 1 mL enzyme per 100 g and *t*=40 °C), 4 (distilled water, time=1 h, 5 mL enzyme per 100 g, *t*=40 °C) and 5 (pH=5, time=1 h, 5 mL enzyme per 100 g, *t*=50 °C) had the highest carotenoid mass fraction on fresh mass basis, expressed as lycopene equivalent ((49.4±1.2), (53.5±0.8) and (55.2±1.4) µg/g, respectively, Fig. 2a). Treatment 5 (pH=5, time=1 h, 5 mL enzyme per 100 g, *t*=50 °C) had the highest antioxidant activity, expressed on fresh mass basis as Trolox equivalent ((1.1±0.5) µmol/g fm) followed by treatments 1 (pH=5, time=1 h, 1 mL enzyme per 100 g and *t*=40 °C), 4 (distilled water, time=1 h, 5 mL enzyme per 100 g, *t*=40 °C) and 6 (distilled water, time=5 h, 1 mL enzyme per 100 g, *t*=50 °C) (Fig. 2b). For both the carotenoid mass fraction and the antioxidant activity of the extracts, the treatment time was the most influential factor, followed by the temperature, the pH of the medium, and finally the volume of enzyme cocktail added per 100 g (ignoring interactions among factors, α=0.05, Fig. S1). This last factor was not significant under the tested conditions.

A fractional factorial design defines, in addition to the significance of the factors, the optimal levels for each factor to maximise the response variables. It is worth mentioning that even if some factors are not significant, as the enzyme amount was, the method selects their optimal level. The conditions of treatment 5 maximised the values of both studied parameters: reaction medium pH=5, reaction time of 1 h, temperature of 50 °C and enzyme volume of 5 mL per 100 g. The optimal pH, temperature and enzyme amount are similar to those reported by Ladole *et al.* (*19*) (pH=5, *t*=50 °C and 3 mL enzyme per 100 g, respectively) but those authors used a shorter enzymatic treatment time (20 min). This difference could be because those authors used oven-dried material, which produces more uniform and smaller particles, while in this study fresh tomatoes were cut, frozen with liquid nitrogen and then pulverized before extraction.

### Optimisation of the extraction by combining environmentally friendly technologies

From experiments 1 and 2, the conditions of enzymatic treatment to be applied in experiment 3 were selected. In this experiment, the enzymatic treatment and later probe sonication combined with green solvents were tested. Moreover, the sonication conditions were assayed previously (data not shown), with the sonication time being the only studied factor (5, 10 and 15 min). From these previous experiments, 10 min of sonication was selected to maximise the carotenoid content and antioxidant activity. Table 2 shows the treatment arrangement.

**Fig. 3:**
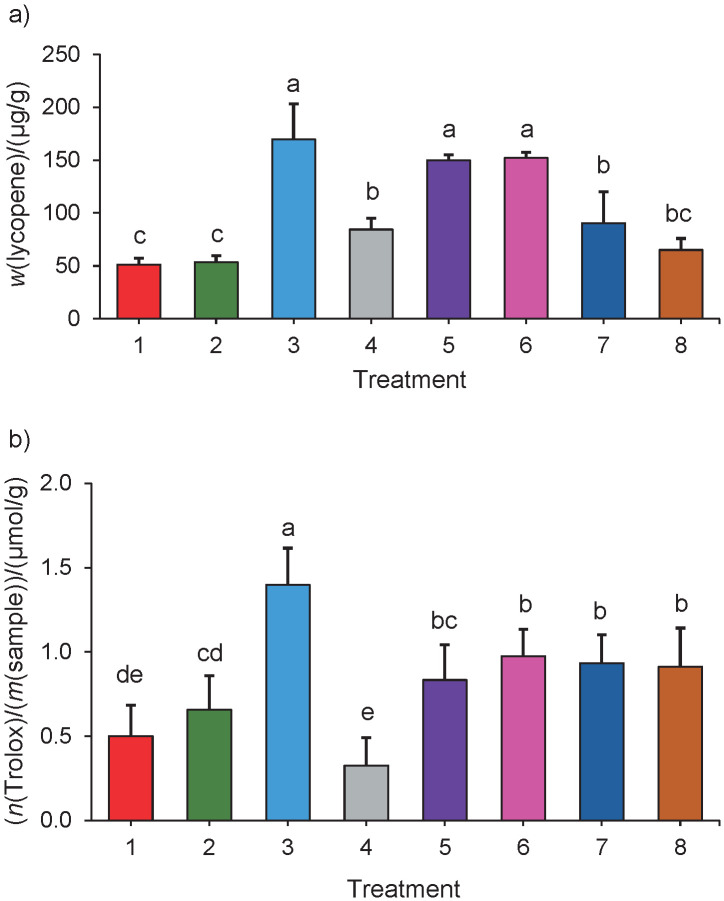
The average values of: a) carotenoid mass fractions, and b) antioxidant activities of extracts obtained from native tomatoes by using KTN enzyme cocktail (with cellulase, glucanase, hemicellulase, pectinase and xylanase activities) and/or sonication (1: 210, sonication, enzyme, ethyl lactate; 2: 210; no sonication, no enzyme, ethyl lactate; 3: 209, sonication, enzyme, isopropyl acetate; 4: 209, no sonication, no enzyme, isopropyl acetate; 5: 210, sonication, no enzyme, isopropyl acetate; 6: 210, no sonication, enzyme, isopropyl acetate; 7: 209, sonication, no enzyme, ethyl acetate; 8: 209, no sonication, enzyme, ethyl acetate). Different letters indicate significant difference according to the Tukey’s test (α=0.05)

The results of experiment 3 are shown in Fig. 3. The carotenoid mass fraction on fresh mass basis, expressed in lycopene equivalents, was similar in treatments 3 (genotype=209, sonication=yes, enzyme=yes, solvent=isopropyl acetate), 5 (genotype=210, sonication=yes, enzyme=no, solvent=isopropyl acetate) and 6 (genotype=210, sonication=no, enzyme=yes, solvent=isopropyl acetate) ((165.3±43.6), (149.5±3.7) and (150.3±4.7) µg/g, respectively) and higher than those of the other treatments. However, the highest antioxidant activity on fresh mass basis, expressed in TE, was found in the extract obtained by treatment 3 ((1.4±0.1) µmol/g). Interestingly, treatment 4 (genotype=209, sonication=no, enzyme=no, solvent=isopropyl acetate) had the lowest antioxidant activity ((0.2±0.1) µmol/g). Unexpectedly, one of the lowest carotenoid mass fractions on fresh mass basis, expressed as lycopene equivalent, was found in treatment 1 (genotype=210, sonication=yes, enzyme=yes, solvent=ethyl lactate; (51.1±0.1) µg/g) despite the enzymatic treatment and sonication. Treatments 3 and 4 using the same tomato genotype had the highest and lowest antioxidant activity, respectively. In addition, two treatments with the application of enzymes and sonication (treatments 1 and 3) had the highest and lowest carotenoid content, respectively. It is important to note that, while in treatments 1 and 3 different solvents were used, in treatments 3 and 4 the same solvent was used. These contradictory behaviours reflect the importance of studying the extraction conditions by using experimental designs and statistics because these factors can significantly modify the properties of the obtained extracts.

Contrary to the results of experiment 2, the studied factors had a different influence on each parameter. While the solvent had the major impact on the carotenoid content (Fig. S2a), the application of digestive enzymes had the most significant influence on the antioxidant activity (Fig. S2b). Interestingly, in the case of the carotenoid content, neither the application of enzymes nor the studied genotype were significant factors, while for the antioxidant activity, the solvent was the factor with the most negligible significance (fractional factorial experiment analysis, α=0.05).

Although the factors had variable relevance according to their effect on the carotenoid content and antioxidant activity, the method indicated the same optimal levels – treatment 3, namely genotype 209, sonication, enzyme treatment and isopropyl acetate – to maximise both responses. Sonication is used to damage tissue integrity by the cavitation phenomenon, in which gas bubbles are formed and grow until they violently collapse, causing implosions that can break cellular walls (*36*). This treatment has been applied successfully to extract lycopene from tomatoes (*37*, *38*). Regarding the solvent, isopropyl acetate and ethyl lactate are green solvents with good results based on previous publications (*31*, *39*). Besides providing better extraction in this work, isopropyl acetate has some advantages over ethyl lactate such as having a lower boiling point, limiting the temperature reached during the sonication and making it easier to obtain a dried extract as well as protecting thermolabile compounds. The two studied tomato genotypes had a similar carotenoid content because the genotype was not a significant factor for this parameter according to statistics (α=0.05, Fig. S2a); however, the antioxidant activity depended on this factor, with genotype 209 exhibiting the highest antioxidant activity.

Due to the reduction in the number of experimental runs in a fractional factorial design compared with complete factorial experiments, the information that can be obtained from them is limited. If there is an interest to know more precisely the effect of intermediate levels of one factor on the results or the effect of interactions among factors and levels, it would be necessary to study that factor in another type of experimental design, such as factorial designs or by using response surface designs (*35*). However, as only two levels were possible for each variable in this experiment, a fractional factorial design was suitable. Besides, Pareto diagrams (Fig. S1 and Fig. S2) are helpful to choose effectively between levels, as well as to decide whether it is necessary to carry out more experiments according to the significance of the factors.

### Quantification of carotenoids and tocopherols in native tomato extracts by HPLC

As experiment 3 was the final experiment (experiments 1 and 2 were preliminary experiments), the extracts obtained in it were characterised in more depth to determine the most abundant carotenoids and tocopherols by HPLC. These results are presented in Table 3.

**Table 3:** Carotenoid and tocopherol mass fractions in native tomato extracts

Treatment	*w*/(µg/g)					
Lycopene	β-carotene	α-tocopherol	β-tocopherol	γ-tocopherol	δ-tocopherol
1	(56.4±5.7)^e^	(1.6±0.2)^c^	(3.2±0.6)^ab^	(1.0±0.1)^c^	(0.27±0.02)^c^	(0.18±0.03)^b^
2	(61.3±3.7)^e^	(0.2±0.1)^d^	(3.9±0.9)^ab^	(1.0±0.1)^c^	(0.45±0.05)^b^	(0.41±0.08)^a^
3	(179.4±15.3)^a^	(20.5±3.9)^a^	(4.2±0.4)^a^	(1.56±0.09)^a^	(0.43±0.09)^b^	(0.29±0.04)^b^
4	(102.5±7.5)^c^	(10.4±3.9)^b^	(2.2±0.3)^b^	(1.1±0.1)^bc^	(0.37±0.06)^b^	(0.04±0.01)^c^
5	(147.6±11.4)^b^	(14.3±5.8)^ab^	(3.2±0.7)^ab^	(1.4±0.2)^ab^	(0.8±0.3)^a^	(0.42±0.06)^a^
6	(114.1±8.2)^c^	(1.8±0.1)^c^	(2.7±0.5)^ab^	(1.2±0.2)^bc^	(1.0±0.2)^a^	(0.34±0.05)^b^
7	(145.7±14.8)^b^	(3.4±1.8)^c^	(4.2±0.6)^a^	(1.1±0.2)^bc^	(0.44±0.04)^b^	(0.27±0.03)^b^
8	(82.6±10.6)^d^	(2.0±0.6)^c^	(3.2±0.4)^ab^	(1.2±0.2)^bc^	(0.4±0.1)^b^	(0.33±0.08)^b^

Results are expressed as mean value±S.D.; *N*=3. Different letters indicate significant differences according to the Tukey’s test, α=0.05

Lycopene was the most abundant carotenoid on fresh mass basis (56.2−179.4 µg/g) followed by β-carotene (0.2−20.5 µg/g). These results are similar to previous reports of red tomato extracts (*40*, *41*). Significant differences between treatments were observed; the treatment with the highest carotenoid mass fraction was treatment 3 (genotype=209, sonication=yes, enzyme=yes, solvent=isopropyl acetate), which is in line with our spectrophotometric results presented in experiment 3. The lower mass fractions, also in accordance with the spectrophotometric results, were found in treatments 1 (genotype=210, sonication=yes, enzyme=yes, solvent=ethyl lactate) and 2 (genotype=210, sonication=no, enzyme=no, solvent=ethyl lactate). In this work, lutein was found only in trace amounts in the extracts, so its values are not presented.

In the literature, it is possible to find very wide ranges of lycopene and β-carotene contents in cherry tomatoes or native tomatoes. Recent works have reported lycopene mass fractions in Mexican native cherry tomatoes in a range of 44−82 µg/g (*7*, *41*). Vela-Hinojosa *et al.* (*41*) also reported a β-carotene content between 1.4 and 3.0 µg/g in cherry red native tomatoes. Kavitha *et al.* (*42*) analysed some cherry and Indian native tomatoes and reported lycopene and β-carotene mass fractions on fresh mass basis between 20.0 and 151.0 µg/g and between 10.0 and 90.0 µg/g, respectively (the latter only reported for cherry genotypes). However, Zanfini *et al.* (*43*) reported lycopene values on fresh mass basis as high as 42.2−60.9 mg/g for Italian native red genotypes, while the cherry Shiren genotype had 184.4 mg/g. In the same work, native genotypes had 8.4−12.4 mg/g of β-carotene and the cherry genotype had 64.8 mg/g of β-carotene; these values are markedly higher than those reported in the present work.

In the extracts obtained by applying non-conventional methods, the four known isoforms of tocopherols were detected. The abundance of them was in the order α>β>γ>δ (Table 3), a finding that is consistent with previous results (*41*). Similarly to the carotenoid content, treatment 3 (genotype=209, sonication=yes, enzyme=yes, solvent=isopropyl acetate) had the highest tocopherol mass fraction on fresh mass basis (α-tocopherol (4.24±0.45) µg/g and total tocopherols (6.5±0.3) µg/g). However, a significant difference was found only *versus* treatment 4 (genotype=209, sonication=no, enzyme=no, solvent=isopropyl acetate), which had the lowest tocopherol mass fraction ((2.2±0.3) µg/g of α-tocopherol and (3.7±0.3) µg/g of total tocopherols). Extracts obtained by treatments 3 and 4 also had the highest and lowest antioxidant activity values, respectively (Fig. 3), suggesting a high contribution of tocopherols to antioxidant activity.

The extract with the highest carotenoid and tocopherol contents also had the highest antioxidant activity (treatment 3); however, the lowest antioxidant activity was found in the extract with the lowest tocopherol content but not the lowest carotenoid content (treatments 4 and 1), suggesting that tocopherols have a better correlation with antioxidant activity than carotenoids. To test this hypothesis, a correlation analysis between the functional compounds and the antioxidant activity of the extracts was carried out (Table 4). The analysis showed that both the carotenoid and tocopherol contents correlated positively with the antioxidant activity, with a better correlation of the tocopherol content (Pearson coefficients 0.66 *versus* 0.83). Interestingly, in these correlations, the lycopene content was correlated with the antioxidant activity (0.68), but the α-tocopherol content did not correlate with antioxidant activity (0.58), these two compounds are the main representatives of carotenoids and tocopherols, respectively. These results make it difficult to conclude about the contribution of each compound or group of compounds to the antioxidant activity, suggesting interactions among different components present in the extracts. As an example of how intricate these antioxidant interactions can be, two studies tested the synergistic effect among carotenoids (including lycopene) and α-tocopherol (*3*, *15*). While Kotíková *et al.* (*3*) reported a non-synergistic interaction among lycopene and α-tocopherol, Zanfini *et al.* (*15*) reported that the lycopene and α-tocopherol mixture had the maximum synergistic effect. These differences could be due to (among other factors) differences in the tested concentrations. Interestingly, Zanfini *et al.* (*15*) also reported no synergistic effects in a lycopene, β-carotene, lutein and α-tocopherol mixture. Another example of positive interactions among carotenoids and tocochromanols was previously published by our group (*39*).

**Table 4:** Correlations between content of carotenoids and tocopherols and antioxidant activity

	Carotenoids	Tocopherols	α-tocopherol	Lycopene	Antioxidant activity
Carotenoids		0.478/0.116	0.260/0.267	0.996/0.001*	0.664/0.036*
Tocopherols	0.478/0.116		0.880/0.002*	0.494/0.107	0.828/0.005*
α-tocopherol	0.260/0.267	0.880/0.002*		0.273/0.257	0.582/0.065
Lycopene	0.996/0.001*	0.494/0.107	0.273/0.257		0.683/0.031*
Antioxidant activity	0.664/0.036*	0.828/0.005*	0.582/0.065	0.683/0.031*	

Results are shown as correlation coefficient/p-value. *Indicates positive significant correlation, α=0.05

## CONCLUSIONS

There are very few reports in which simple and environmentally friendly extraction methods, including green solvents, are combined to obtain antioxidants from tomato. The use of various experimental stages provided a more comprehensive study of the extraction process. Preliminary experiments – experiments 1 and 2 in this study – are always required to test the performance of enzyme cocktails and to establish optimal enzyme cocktail conditions to ensure the maximum yield of carotenoids and antioxidant activity, because each vegetal material has a distinct behaviour. Experiment 1 showed that the enzyme cocktail containing pectinase had the best performance regarding the carotenoid content and antioxidant activity. In experiment 2, the conditions for the enzyme cocktail reaction were optimised by using a fractional factorial design to save resources, increasing the efficiency of the extraction procedure. Experiment 3 was performed to test the effect of the combination of the enzyme cocktail and sonication. A significant increase of approx. 40 to 100% of carotenoids, 30 to 100% of tocopherols, and 20 to 400% of antioxidant activity was observed compared to the extraction method without sonication and without enzymes. Interestingly, antioxidant activity had a significant positive correlation with lycopene, total carotenoids and total tocopherols. Taken together, these results indicate that the antioxidant activity of the lipophilic tomato extracts can be enhanced by applying a combination of environmentally friendly extraction methods, presumably by increasing the amount of both carotenoids and tocopherols. Since the antioxidant activity of tomato extracts is a result of the interaction among all their components, those methods that increase the extraction of all lipophilic antioxidants are preferable to single compound target methods.
